# Radiology assessment of omphalopagus conjoined twins: A case report^☆^

**DOI:** 10.1016/j.radcr.2022.01.042

**Published:** 2022-02-04

**Authors:** Syahriar Muhammad, Budi Laraswati, Lenny Violetta

**Affiliations:** Radiology Resident, Department of Radiology, Faculty of Medicine Universitas Airlangga - Dr Soetomo Academic General Hospital, Surabaya, Indonesia

**Keywords:** Conjoined Twins, Omphalopagus, CT Scan, Echocardiography, Imaging

## Abstract

Conjoined twins are rare and present a challenge for surgeons and radiologists and classified according to the main site of connection: thorax (thoracopagus), abdomen (omphalopagus), etc. Here, we report a Seventeen-month-old, female omphalopagus conjoined twins, born from a mother with a family history of twins, who performed CT-scan and ultrasound echocardiography for elective surgery preparation and X-ray evaluation after the separation surgery. From the CT-Scan examination, revealed each baby had its own, separate heart (one with dextrocardia), each baby had its own liver but they were partially fused and several small branches crossed each other (superior mesenteric artery, intercostal artery, and hepatic vasculature). Findings at surgery are consistent with radiological findings, but we missed to evaluate the pericardium despite being informed by the CT-scan that each baby had its own heart. Radiological investigation plays an important role in the evaluation, all possibilities must be taken into account: operation feasibility, shared organs, soft tissue, and bone structure.

## Introduction

Conjoined twins are a very rare developmental anomaly of uncertain aetiology, and the prevalence varies widely from 1:50,000 to 1:200,000 [1,2]. From 40% to 60% of conjoined twins are stillborn, and almost 35% of live births do not survive past 24 hours [1]. Conjoined twins are monozygotic, monoamniotic, and monochorionic, and are always of the same gender with a 3:1 female preponderance. Embryologically, their formation results either from failure of separation of the embryonic plate between day 15 and 17 of gestation, or from secondary union of 2 separate embryonic discs at the dorsal neural tube or ventral yolk sac areas from week 3-4 of gestation [3], [4], [10]. Conjoined twins are classified according to the most prominent site of connection: the thorax (thoracopagus), abdomen (omphalopagus), sacrum (pygopagus), pelvis (ischiopagus), parapagus (fused side-by-side with a shared pelvis), skull (craniopagus), face (cephalopagus), or back (rachipagus). Thoracic conjunction is most common and requires cardiac assessment. MRI and CT-scan provide excellent anatomic and bone detail, demonstrating organ position, shared viscera, and limited vascular anatomy. Contrast radiography allows for evaluation of the gastrointestinal and urogenital tracts, and a shared liver requires assessment of anatomy, vascularisation, and biliary drainage. Angiography helps to define specific vascular supply, which is useful in determining the distribution of shared structures between the twins at the time of surgery [5]. Surgical outcomes are better in twins who do not share vital organs such as the heart or brain, and the best results are for omphalopagus twins.

**Fig. 1:**
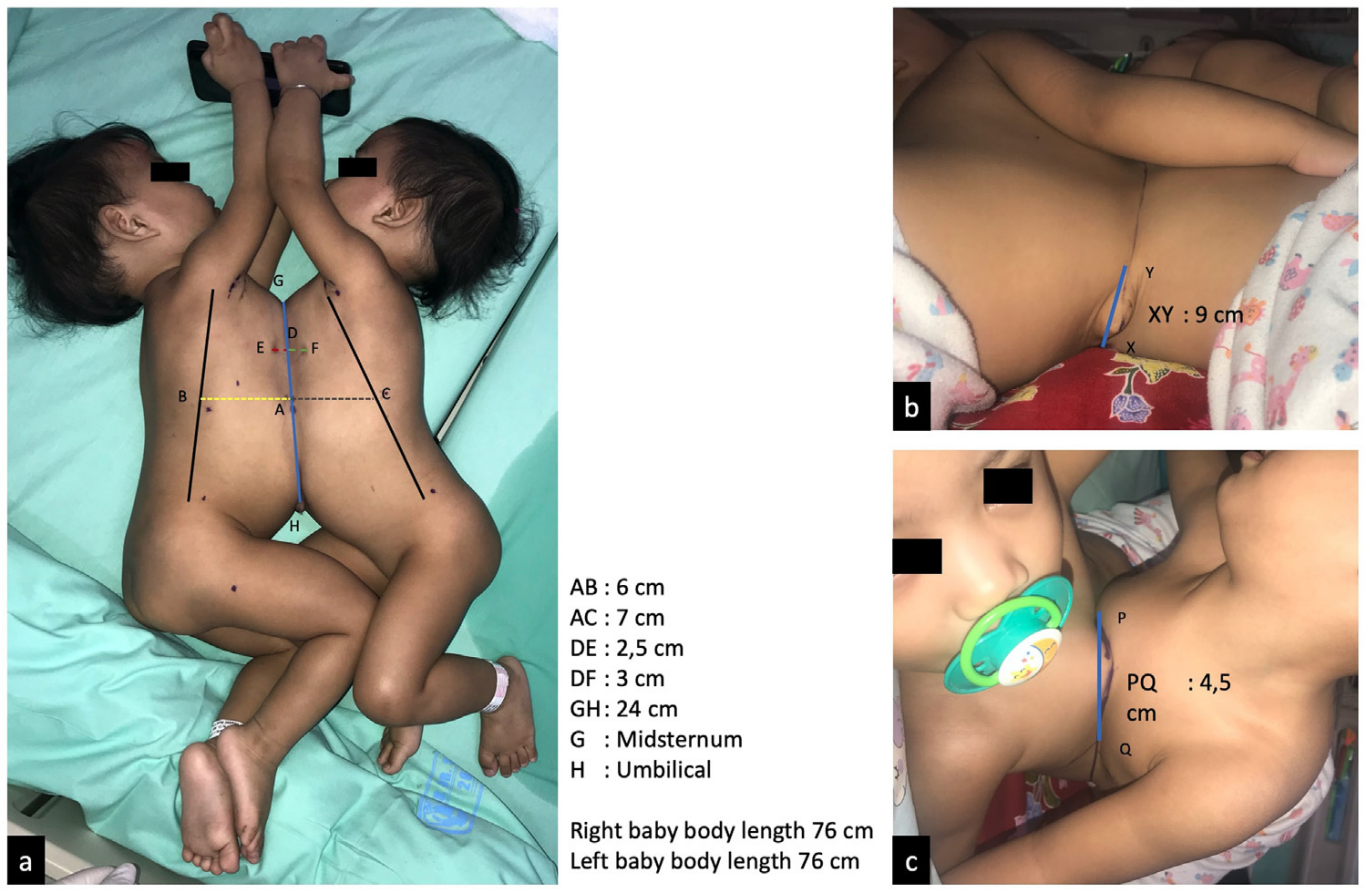
Physical appearance of the conjoined twins at 17 months of age, with skin surface measurements for the surgical area.

## Case descriptions

Seventeen-month-old, female conjoined twins were referred from the Regional Public Hospital in Kendari, South-East Sulawesi Province, to Dr Soetomo Academic General Hospital, Surabaya, East Java Province, Indonesia. The patients’ mother was a 20-year-old woman with a family history of twins. Evidence of the conjoined twins was detected during the antenatal ultrasound. The first CT-scan was performed at the Regional Public Hospital on March 9, 2018, when the patients were 2 days old, and the following was observed: the position of the liver appeared dominant in the left baby's sub-diaphragm (possibly a single liver); both right and left babies’ bowel loops were normal, separated, but seemed fused and/or connected at the umbilical area; and pneumonia was observed in the left baby. Because the patients did not require immediate surgery, surgery was not performed at that time, and the patients’ parents followed the doctor's advice to prepare the patients for elective surgery.

**Fig. 2:**
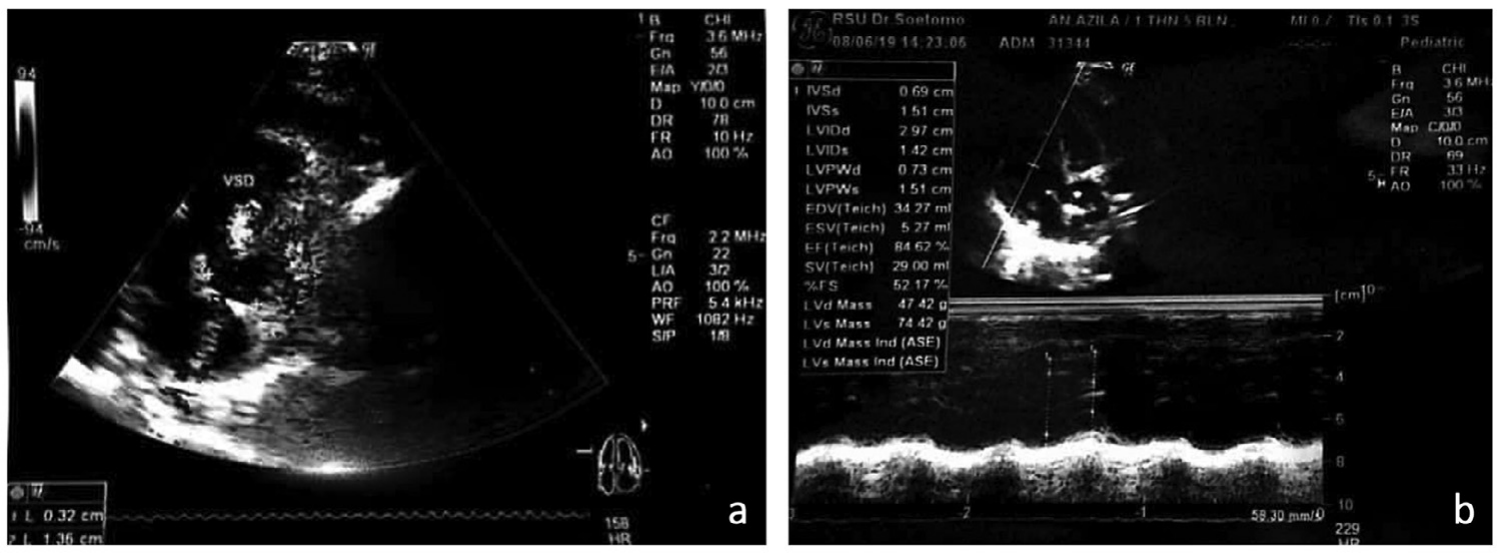
Echocardiography performed on August 6, 2019. (a) In the right baby, a small perimembranous VSD with diameter ± 0,32 cm was detected; (b) the left baby had normal echocardiography results.

At the age of 17 months, the patients were referred to Dr Soetomo Academic General Hospital, Surabaya. The patients were treated by the conjoined twin's medical team and were prepared in advance to minimise surgical complications (Fig. 1). For radiological imaging, both right and left babies underwent echocardiography and CT-scan with contrast. Echocardiography detected a small perimembranous ventricular septal defect (VSD) in the right baby (Fig. 2).

**Fig. 3:**
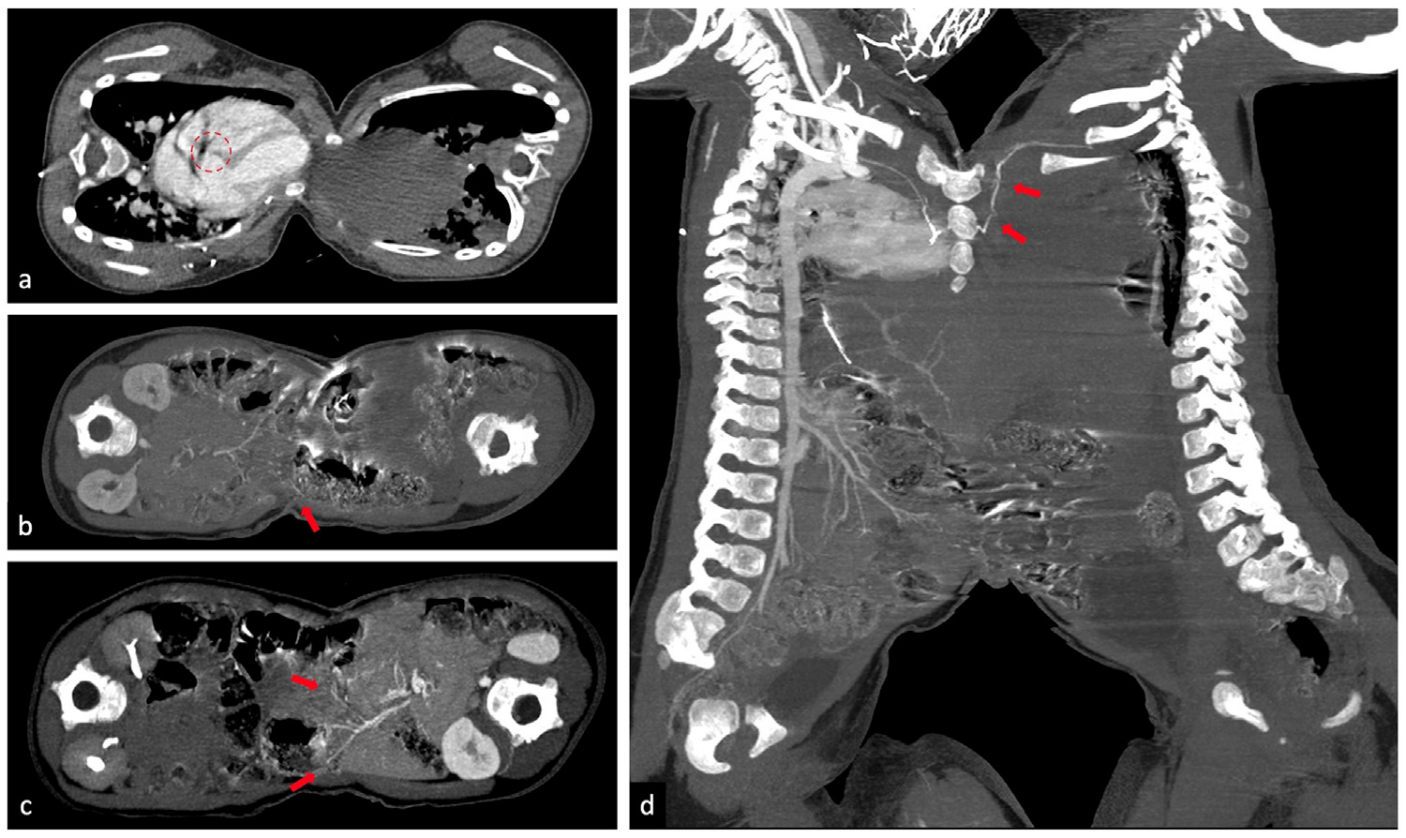
(a) Arterial phase of right baby, contrast material is confined to the cardiac chambers only in the right baby, which establishes that there is no vascular connection between the 2 hearts, making these twins omphalopagus rather than thoraco-omphalopagus, but there is small VSD diameter ± 0,32 cm (dashed red circle); (b) arterial phase of right baby; (c) arterial phase of left baby, showing some small branches of the superior mesenteric artery of the right and left babies crossing in the midline (red arrow); (d) arterial phase of the right baby, branches from the intercostal artery at the level of the manubrium of sternum from the right baby enter the vascular system of the left baby (red arrow), and sternal fusion as high as the manubrium of sternum from the right baby (left baby does not have a sternum) (Color version of the figure is available online.)

**Fig. 4:**
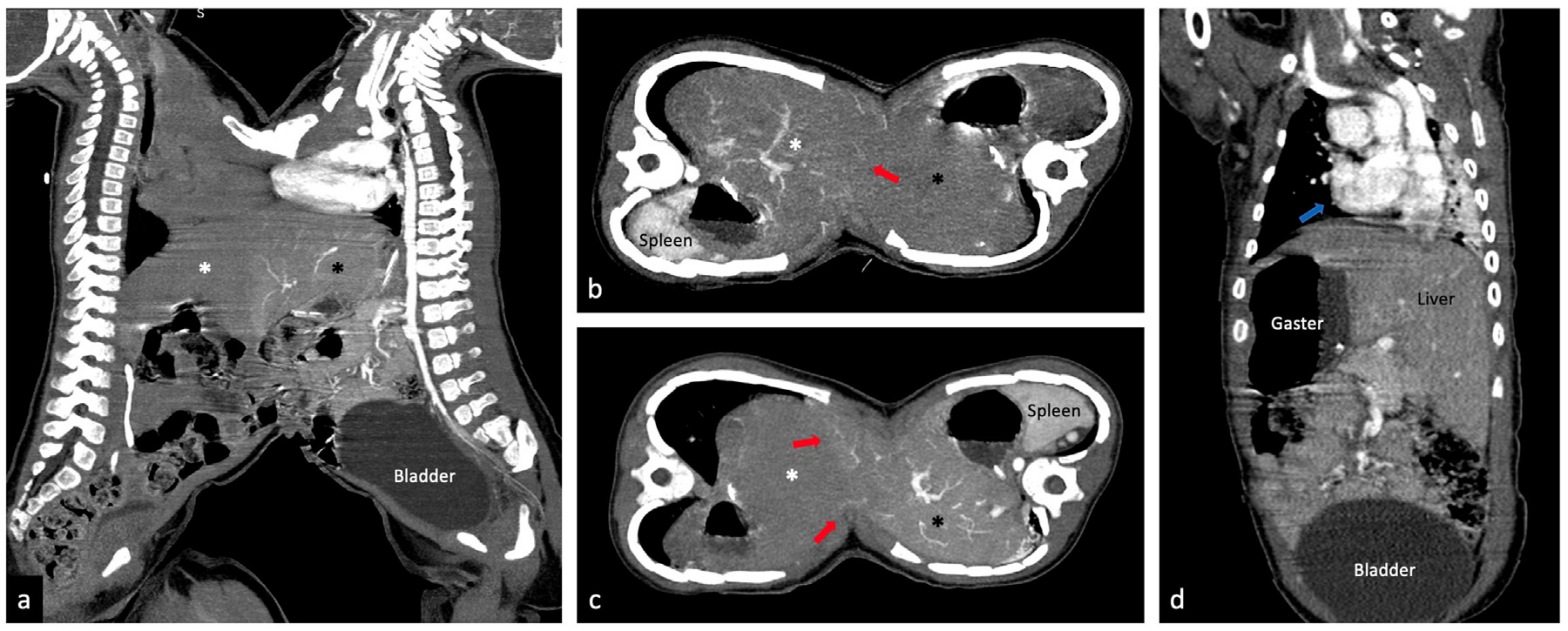
(a,b,c) Each baby has its own liver but they are partially fused and small branches of the hepatic vasculature seem to cross in the midline (red arrow); (d) left baby coronal view, left baby has dextrocardia (apex of heart at the right side, blue arrow) with situs inversus (Color version of the figure is available online.)

**Fig. 5:**
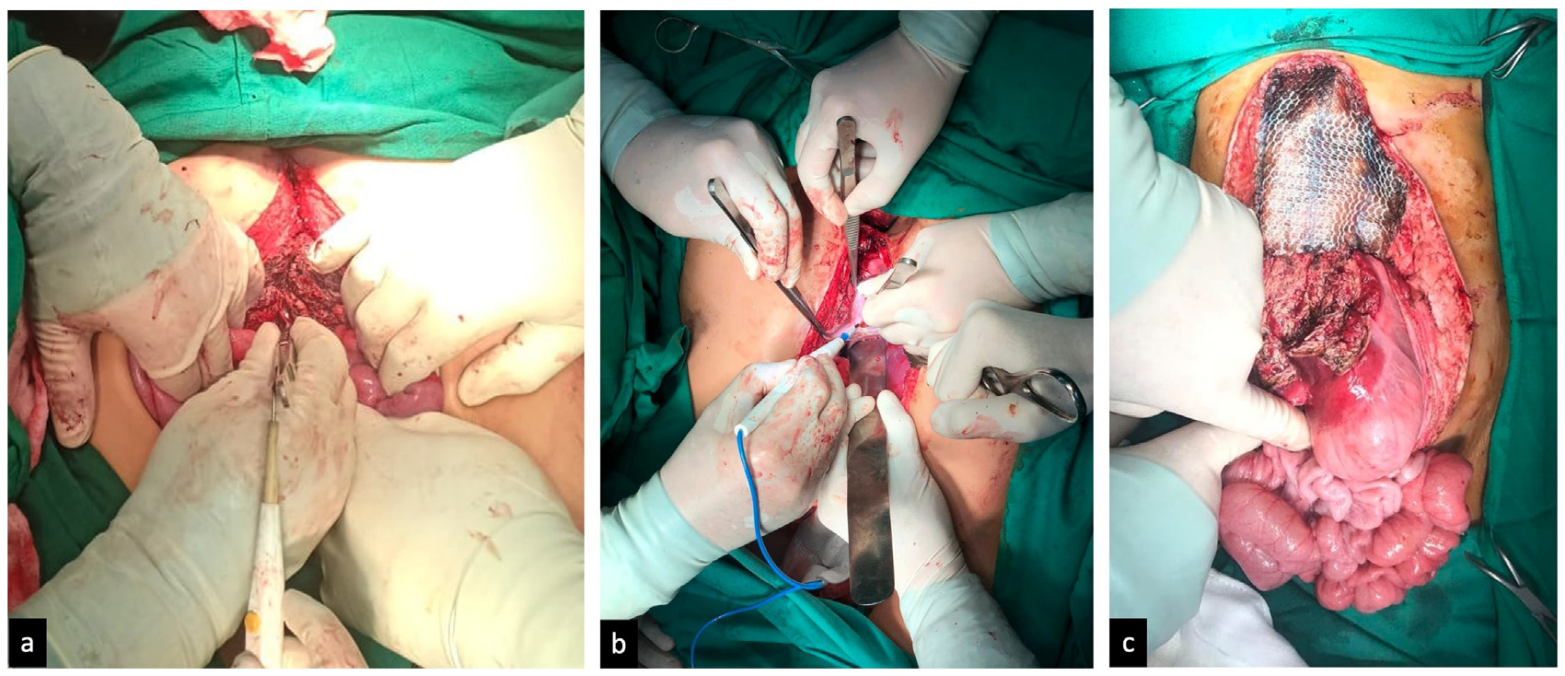
Operation performed on August 14, 2019. (a) Separation of the livers; (b) separation of the sternum and pericardium; (c) small and large bowels are normal.

CT-scan examination on August 5, 2019 (the CT-scan was performed feet-first using an indirect scanning procedure because there were many medical devices attached to the patients and to make it easier for anaesthesiologist to perform sedation or anaesthesia) revealed the following: thoracoabdominal fusion from as high as the second thoracic vertebra to the fifth lumbar vertebra, with fusion measuring ± 18,6 × 9,9 cm from the outer of skin and ± 17,6 × 9,1 cm from the inner subcutis; the left baby had dextrocardia with situs inversus (Fig. 4d); each baby had its own, separate heart; the fat line was (+), making these twins omphalopagus rather than thoracopagus; several small branches of the superior mesenteric artery of the right and left babies crossed the midline and branches from the right baby's intercostal artery at the level of the manubrium of the sternum entered the vascular system of the left baby (Fig. 3); each baby had its own liver but they were partially fused, and small branches of the hepatic vasculature seemed to cross in the midline (Fig. 4); sternal fusion was observed as high as the manubrium of sternum that seemed to originate from the right baby (the left baby did not have a sternum) (Fig. 3d); and a second thoracic butterfly vertebra in the left baby. Each baby had its own pancreas, spleen, 2 kidneys, and normal urinary tract and bladder. The separation operation procedure are shown in (Fig. 5).

**Fig. 6:**
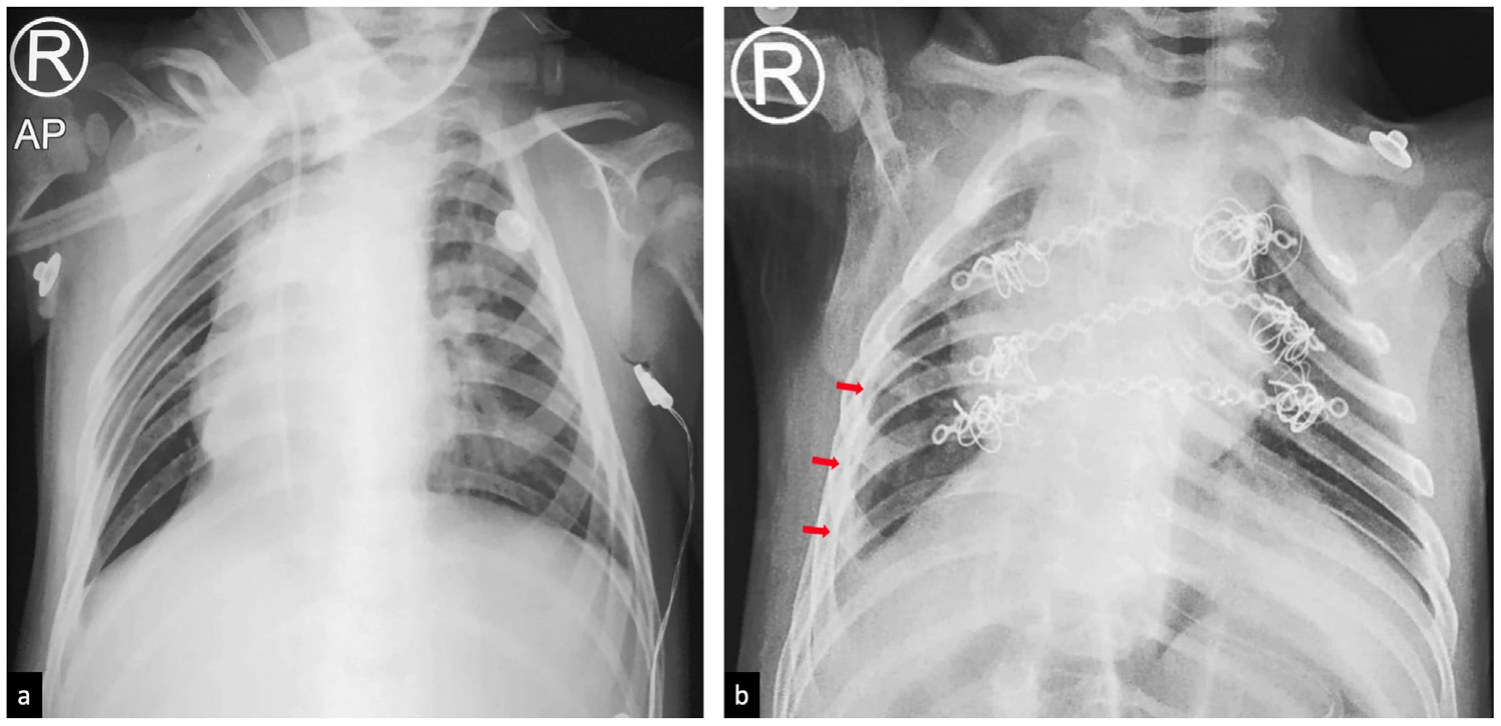
Post-surgery X-ray evaluations. (a) Four days after surgery, AP projection chest X-ray (rotated) of the right baby appears normal; (b) nineteen days after surgery, AP projection chest X-ray of the left baby shows dextrocardia (heart apex at right side) and laminar pleural effusion (red arrow). The left baby did not have a sternum and temporary internal fixation was placed to allow the lung to expand properly; when she is older, it will be replaced with a proper implant (Color version of the figure is available online.)

After the operation, the separated babies remained under observation in the intensive care unit and chest x-rays was taken on both babies for evaluation (Fig. 6). The right baby's recovery process was faster than the left baby's, and she spent only 1 week in the intensive care unit before being transferred to the paediatric ward to await her twin's recovery. After 1 month in the intensive care unit, the left baby's recovery process was considered adequate and she was moved to paediatric ward and observed for 2 weeks. Both patients are healthy at the time of submission of this case report.

## Discussion

Conjoined twins are rare and present a challenge to surgeons and radiologists [5]. In cases of conjoined twins, we must determine the priority and whether the case is life-saving or not, then determine whether separation surgery can be performed. If separation cannot be completed, the patients are treated conservatively without attempting surgical separation. The indications for emergency surgery include the following: one twin is stillborn or has anomalies incompatible with life; damage to the connecting bridge; ruptured omphalocele or other life-threatening event; and congenital anomalies that are surgically correctable but would be fatal if not treated [5]. In some cases, there are ethical considerations as the survival of both twins is unlikely and one twin may have to be sacrificed to save the other [6]. In emergency situations, the twins are in unstable condition and plain radiography, echocardiography, and ultrasound (head and abdominal) are usually performed as bedside examinations in the neonatal intensive care unit. If the patients are stable and operable, they are prepared optimally. The ideal treatment is elective surgery at 6–12 months of age to allow time for growth, tissue expansion, and adequate imaging to accurately demonstrate the anatomic union and associated anomalies to aid surgical planning [7]. Although omphalopagus twins have the best chances of survival, solid team management, preoperative and postoperative planning are required [8]. All possibilities must be taken into account: operation feasibility, shared organs, soft tissue, and bone structure. There are many possibilities that can happen after surgery.

Radiological investigation plays an important role in the evaluation of shared organs, anomalies, and the presence and extent of cross circulation [9]. In our case, CT-scan was performed feet first and with an indirect scanning procedure because there were many medical devices attached to the patient and this method made it easier for the anaesthesiologist to perform sedation or anaesthesia. We performed a 3-phase thoracoabdominal CT-scan (arterial, venous, delayed for 10 minutes) for each baby to evaluate vasculature, shared organs, the pelvicalyceal systems, and urinary bladders. In this case, we missed to evaluate the pericardium despite being informed by the CT-scan that each baby had its own heart. Even when the hearts are separated, echocardiography should be performed to evaluate accompanying congenital heart diseases such as ASD or VSD; if echocardiography has previously revealed these abnormalities, we can still evaluate whether the defect has closed or widened. It is sometimes difficult to determine whether the bowels are separate or fused from the CT-scan. Barium follow-through or barium enema can be performed for better evaluation of the bowels, especially in omphalopagus conjoined twins [5].

## Conclusion

Every case of conjoined twins is rare and unique; therefore, an imaging strategy to accurately define anatomic fusion, vascular anomalies, and other related abnormalities is important for surgical planning and the collection of prognostic information.

## Consent and ethic committee approval

Written consent has been obtained from the patients as there is no patients identifiable data included in this case report. This study has met the ethical principle and already got approval from Research Ethics Committee from Dr Soetomo General Hospital, Surabaya.

## Patient consent

*Informed consent obtained for publication of a case report:*

Written informed consent was obtained from patients for the publication of this case report.
